# COMP Is a Biomarker of Cartilage Destruction, Extracellular Matrix and Vascular Remodeling and Tissue Repair

**DOI:** 10.3390/ijms26189182

**Published:** 2025-09-19

**Authors:** Margaret M. Smith, James Melrose

**Affiliations:** 1 Raymond Purves Bone and Joint Research Laboratories, Kolling Institute, Faculty of Medicine and Health, The University of Sydney and the Northern Sydney Local Health District, Sydney, NSW 2065, Australia; mobsmith@sydney.edu.au; 2 Arthropharm Australia Pharmaceuticals Pty Ltd., Bondi Junction, Sydney, NSW 2022, Australia; 3 Graduate School of Biomedical Engineering, University of New South Wales, Sydney, NSW 2052, Australia

**Keywords:** COMP, biomarker, chondrogenesis, osteogenesis, OA, cartilage breakdown, repair biology

## Abstract

This review covers the roles of cartilage oligomeric matrix protein (COMP), an established biomarker of cartilage breakdown in pathological tissues in osteoarthritis, and in emerging areas in extracellular matrix and vascular remodeling associated with trauma, fibrosis and cancer. COMP is produced by chondrocytes, tenocytes, myofibroblasts, and in some specialized tissue contexts, endothelial and vascular smooth muscle cells. COMP expression by tendon and cartilage cells is sensitive to weight bearing and tensional mechanical stimulation. Vascular smooth muscle cells are sensitive to shear forces which regulate COMP expression in vascular tissues in atherosclerosis and in carotid stenosis. COMP is a multivalent bridging molecule that stabilizes tissues. It facilitates the signaling of TGF-β and BMP-2 in chondrogenesis, osteogenesis, tissue fibrosis, vascular and ECM remodeling and tumor development by providing a multimeric environment through which growth factor binding and receptor activation can occur. Engineered COMP proteins have been used as molecular templates in the development of chimeric therapeutic proteins of potential application in repair biology. Tie2 (Angiopoietin-1 receptor, Tyrosine-protein kinase receptor TEK), when activated by an engineered COMP-inspired angiopoietin-2 pentamer, is a potent angiogenic molecule of obvious application in wound healing. COMP’s multifunctional properties show it is much more than a biomolecular marker protein through its ability to participate in many biological processes. Further studies are warranted to fully explore the biology of this fascinating molecule, particularly in the wound repair processes.

## 1. Introduction

Cartilage oligomeric matrix protein (COMP), also known as thrombospondin-5 (TSP-5), is an extracellular matrix (ECM) glycoprotein with critical roles in collagen and ECM assembly and consequently matrix stabilization [1,2,3,4,5]. The aim of this study was to examine the roles of COMP in cartilage but also in tendon and vascular tissues, in tissue fibrosis and in cancer. COMP’s varied roles in these tissues indicate that it has extensive roles in tissue stabilization and function other than just as a stabilizing factor in cartilage. Furthermore, the specific molecular architecture of COMP as a pentameric platform and its interactive properties with TGF-β and BMP-2 in a multimeric environment and dynamic conformational changes in COMP structure upon growth factor binding are conducive not only to growth factor binding but also to receptor activation. Furthermore, engineering of COMP-Ang 1 and COMP-Ang 2 chimeric proteins has produced angiogenic stimulatory molecules with improved biological activity compared to free Ang 1 and Ang 2, with added capability in receptor activation that makes them highly capable of promoting the development of new capillary vessels (blood and lymphatic vessels) that promote wound repair. It is our proposal therefore that COMP should be considered as a multifunctional protein capable of promoting tissue repair processes rather than merely being considered as a biomarker of tissue pathology. Moreover, COMP may also have a role in the regulation of the complement system, providing a connection with the innate immune system. This possibility needs further studies for confirmation but supports our proposal of the multifunctional properties of COMP in health and disease.

### 1.1. COMP and Matrix Stabilization

The complexities of the structure and function of the ECM, where roles for COMP in tissue stabilization have been delineated, have been covered by a number of excellent reviews; the reader is referred to these for further background information [6,7,8,9,10]. COMP/TSP5 is the fifth member of the thrombospondin family, identified in 1992 as a large 550 kDa structural acidic cartilage glycoprotein [11], N-glycosylated at Asn-101 and Asn-721 [12]. COMP shows high-affinity binding to matrilin-1, 3, and 4 [13,14] and to the GXKGHR motif in collagens I and II to promote collagen fibrillogenesis [15,16]. COMP also forms heteropentamers with thrombospondin-4 (TSP4) [17]. While originally believed to be a cartilage-specific protein, studies have now shown COMP is also highly expressed in tendon [18], and its expression [19] and fragmentation can be modulated by intrinsic biomechanical forces carried by the tendon [20,21]. Compressed articular cartilage also has increased TSP-5/COMP transcript levels, and its synthesis by chondrocytes is also sensitive to mechanical loading [22]. COMP synthesis by chondrocytes or tenocytes is thus responsive to the intrinsic biomechanical environment of these cells [23].

**Figure 1:**
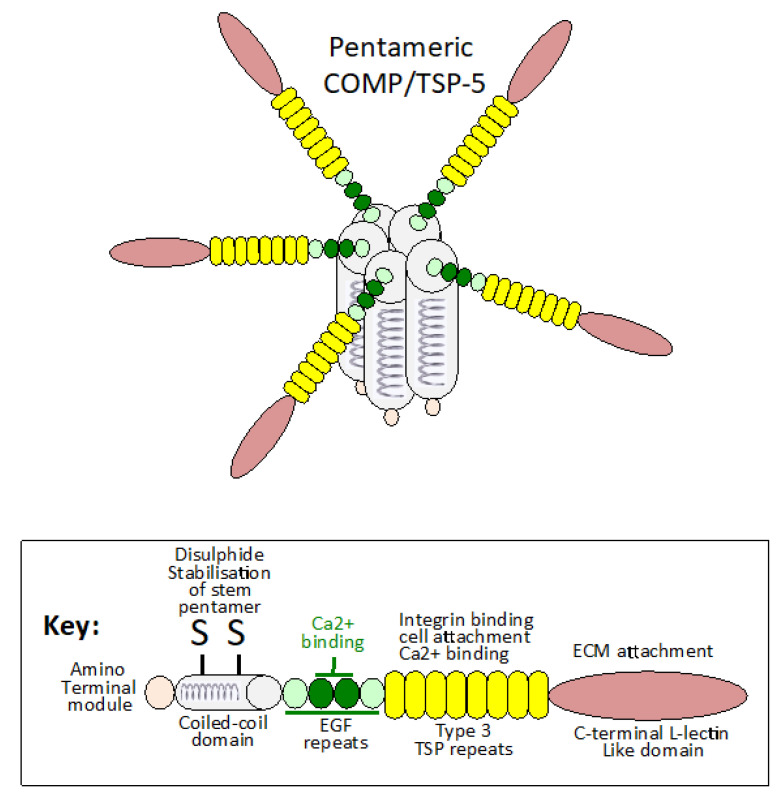
Schematic representation of the COMP pentameric structure showing the disulphide linked coiled-coil domains, which form the stalk from which the COMP monomers radiate away in a flower-head like arrangement with important interactive properties with other ECM components. The two central EGF domains in COMP have predicted Ca^2+^ binding activity that may induce conformational changes in the COMP molecule conducive to its interactions with the GAG side chains of aggrecan, SLRPs and HS-proteoglycans influencing ECM assembly, ECM stabilization and tissue function. Tenocytes and chondrocytes are sensitive to mechanical stimulation, which promotes COMP synthesis, demonstrating important cell–matrix communication that regulates tissue homeostasis. The structure for COMP shown is based on data presented in [30,31].

The modular bridging structure of COMP facilitates interaction with multiple cartilage ECM components, such as collagens I, II, IX, XII, XIV, fibronectin, matrilins-1, 3, 4, and proteoglycans aiding in ECM stabilization [24]. The pentameric structure of COMP also acts as a multimeric ligand platform for the presentation of growth factors to cells. COMP acts as a scaffold for growth factors, controlling how and when the growth factors are presented to cell-surface receptors; such multimeric interactions also promote receptor activation. The bouquet-like radial presentation of the pentameric COMP chains thus equips COMP with novel interactive roles in the regulation of a number of cell-signaling pathways that maintain tissue homeostasis and function in health and disease [25] (Figure 1). COMP interacts with TGF-β and the BMP family (BMP-2, 4, 7). In addition to its roles as a biomarker of cartilage destruction, COMP is also a serum biomarker that can be an indicator of cancer progression/poor prognosis and organ/tissue fibrosis [26]. COMP regulates fibrillar collagen assembly. Elevated expression of COMP by skin fibroblasts occurs in systemic sclerosis, keloid formation and in scleroderma [27,28,29]. COMP expression is highest in large keloids (>10 cm^2^) [28]. TGF-β signaling is critical for skin fibrosis and promoted by COMP, which induces ECM deposition by skin fibroblasts [27].

High-resolution atomic force microscopy (hrAFM) has demonstrated TGF-β1 and COMP complexes containing one to three COMP and multiple TGF-β1 molecules; such multivalent presentation of growth factors may explain COMP’s unique properties in growth factor presentation and the activation of receptors operative in chondrogenesis, osteogenesis, tissue fibrosis and tumor biology [32,33,34,35,36,37,38]. COMP–BMP-2 complexes enhance osteogenesis by osteoprogenitor cells forming ligand–receptor clusters, resulting in sustained activation of the Smad signaling pathway and enhanced osteogenesis [34]. Smad is an acronym from the fusion of *Caenorhabditis elegans* Sma genes and the *Drosophila* Mothers against decapentaplegic Mad genes that promote TGF-β cell signaling.

**Figure 2:**
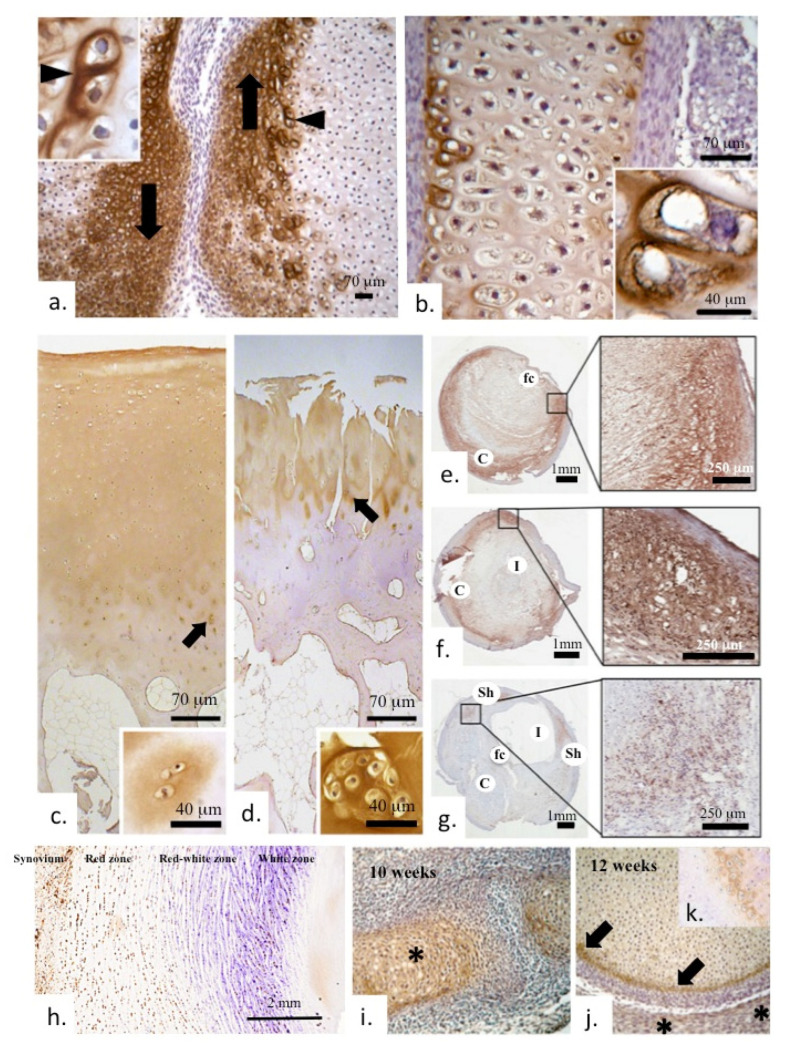
Immunolocalization of COMP in early human bone, cartilage in diarthrodial joint development and in meniscus and plaque formations in carotid blood vessels (**a**). Marked localization of COMP in the margins of the articulating surface regions adjacent to the developing joint space (arrows) in a 10-week gestational age human fetal knee. The inset depicts strong localization of COMP around hypertrophic cells in the region depicted by the arrowhead. (**b**) Prominent COMP immunolocalization in the bone femoral diaphysis of a 12-week gestational age knee specimen; the inset shows prominent pericellular localization. (**c**) Low-power immunolocalization of COMP in full depth normal adult articular cartilage and (**d**) its depletion in OA cartilage with prominent surface fibrillation. Arrowed regions in (**c**,**d**) are shown at higher magnification in the adjacent photo-insets. COMP is seen in the interterritorial matrix of the superficial and middle zones of healthy cartilage, whereas in the deeper zones, a more pericellular distribution is seen (insets). In late-stage OA cartilage, staining is depleted overall and mainly confined to clusters of cells (**d**). COMP is found localized in plaque formations in vascular tissues (carotid artery) (**e**–**g**). Abbreviations used, C, plaque core; fc, fibrous caps; l, lumen; sh, shoulder regions. The indicated boxed regions are shown in adjacent magnified regions. In meniscal tissues, COMP is predominantly localized to the synovium and cartilaginous meniscal red zone (**h**). Low-power immunolocalization in early joint development shows COMP localized in the developing bony elements of the joint at 10 weeks of growth (**i**); by 12 weeks, COMP is mainly confined to the surface of the developing epiphysis (arrows), whereas the developing acetabulum shows less staining (asterisks) (**j**). Inset (**k**) shows COMP immunolocalization in a tibial long bone growth plate. Images reproduced from [77] (plates **a**–**d**,**i**–**k**) and [69] (plates **e**–**g**) under an open access Creative Commons Attribution (CC-BY) license.

COMP participates in cell signaling through CD36 [39], Jagged-1 [40,41], Notch-3 [40,42], Piezo-1 [43] and angiotensin receptor-1 [44] not only in the promotion of chondrogenesis but also in bone formation, skeletal development and vascular repair [45]. COMP regulates BMP-2 signaling in mesenchymal cells to modulate chondrogenesis [46] and stimulates adhesion and motility of vascular smooth muscle cells [47]. Ang1/Tie2 signaling regulates the maintenance of vascular quiescence and promotion of angiogenesis [48]. Ang1 also promotes lymphatic vessel endothelial hyaluronan receptor 1 (LYVE-1) positive vessel development [49]. Lymphatic vessels have roles in tissue homeostasis and immune regulation, aiding in the recovery of normal tissue functions [50]. Engineered COMP-Ang 1 chimeric proteins have been developed with improved angiogenic properties in vascular repair processes [51,52]. COMP-Ang1 chimeras enhance BMP2-induced osteoblast differentiation and bone formation [45]. COMP is a multifunctional protein of diverse functions in chondrocyte biology and skeletogenesis, and is a well-established biomarker of cartilage pathology (Figure 1 and Figure 2). COMP has been used as a biomarker for idiopathic pulmonary fibrosis [53] and cartilage degeneration in OA and RA [54,55] and traumatic joint injury [3,56,57,58] (Table 1).

**Table 1:** Serum COMP As A Discriminative Biomarker Of Cartilage Destruction.

Study	Features	Ref
Clark et al., 1999	Serum COMP levels can distinguish OA from a normal unaffected subgroup, reflecting OA disease severity and the involvement of multiple tissues in the knee joint OA process. The focus should not just be on the articular cartilage. Knee OA is a global disease, and the synovium, meniscus, ligaments, subchondral bone and infrapatellar fat pad all have roles to play in the disease process.	[59]
Vilim et al., 2001	Serum COMP is a measure of synovitis in knee OA. Elevated serum COMP levels in OA patients have been correlated with clinical joint examinations confirming synovitis and changes in other joint tissues.	[60]
Vilim et al., 2002	Correlation of serum COMP levels with radiographic progression of knee OA. Serum COMP levels are a prognostic marker of progressive joint disease and have been shown to persist over a 3-year study period.	[61]
Wisłowska et al., 2005	Serum COMP levels correlate with the severity of systemic lupus erythematosus and knee OA. In systemic lupus erythematosus patients (*n* = 30), serum COMP levels are significantly higher (*p* < 0.05) than serum from normal non OA affected patients (*n* = 30). This demonstrates the variable involvement of inflammation in disease processes in some sub-types of knee joint OA.	[62]
Andersson et al., 2006	Serum COMP levels increased temporarily after physical exercise in 58 patients with knee OA. Exercise increases serum COMP levels in individuals affected with knee OA, COMP levels were decreased during rest. The increased serum COMP levels were normalized 30 min after an exercise session. This demonstrates the dynamic nature of COMP release from knee joint tissues.	[63]
Fernandes et al., 2007	Correlation of serum COMP levels with clinical and radiological knee OA in a Brazilian population. Patients with symptomatic knee OA had significantly higher serum COMP levels than healthy non-OA affected controls or non-symptomatic knees that showed radiographic evidence of narrowing of the joint space. This shows the potential of COMP as a prognostic and diagnostic factor in knee joint OA.	[64]
Tseng et al., 2009	Serum COMP is a marker of knee arthritis and a biomarker of ECM changes following joint trauma or cartilage degeneration. COMP is a diagnostic and prognostic marker of OA severity and can be used to assess the efficacy of anti-arthritic drugs in prospective OA treatments.	[1]
Hoch et al., 2011	Elevation of serum COMP in patients with knee OA: meta-analysis. A meta-analysis of a number of studies which examined serum COMP levels in knees with radiographically diagnosed OA of variable severity showed serum COMP levels were consistently elevated in patients with knee OA and were sensitive to OA disease progression. This confirmed that COMP is a biomarker for OA development and progression.	[55]
Zivanović et al., 2011	COMP, an inflammation biomarker in knee OA. Measurement of serum COMP levels in 88 OA patients examined by ultrasound to assess severity of OA disease and presence of synovitis showed serum COMP levels ranging from 52 to 66.5 ng/mL and correlated with the clinical severity of OA and the involvement of synovitis in knee OA. COMP thus was a biomarker of the severity of inflammation in knee OA.	[5]
Verma et al., 2013	Serum COMP is a novel diagnostic and prognostic biomarker of knee OA. Measurement of serum COMP and inflammatory cytokine levels in OA and normal control patients by ELISA demonstrated COMP levels in OA patients were 1117.21 ng/mL (125.03–4209.75 ng/mL) compared to 338.62 ng/mL (118–589 ng/mL) in control subjects (*p* < 0.001). COMP levels positively correlated with the clinical severity of OA cases and demonstrated COMP was a quantitative biomarker of knee OA.	[4]
Lotz et al., 2013	Current status and perspectives of OA biomarkers. OA biomarkers have been classified into burden of disease, investigative, prognostic, efficacy of intervention, diagnostic and safety categories. Serum COMP as a biomarker falls into the burden of disease, prognostic and diagnostic categories.	[65]
Kluzek et al., 2015	Serum COMP in the development of radiographic, painful knee OA in a community-based cohort of middle-aged women. A study of serum COMP levels in a group of 593 middle-aged women in the development of radiographically diagnosed painful knee OA demonstrated that serum COMP levels were predictive of structural knee-joint tissue changes and the incidence of painful knee OA, independently of age and BMI.	[56]
Henroitin et al., 2016	Current status of cartilage ECM OA biomarkers. Soluble cartilage biomarkers such as COMP have been proposed to be complementary drug development tools useful in the discovery of anti-arthritic drugs from preclinical stages of their development up to their evaluation in the clinic. Such biomarkers should be considered surrogate indicators of clinical and/or imaging outcomes. Use of automated assays for biomarker panels may eventually lead to personalized medicines for enhanced management of OA.	[54]
Ben Achour et al., 2018	Correlation of bone and cartilage biomarkers with structural damage in RA: Cross sectional study. COMP is a biomarker of cartilage destruction that has been shown to be associated with joint erosions characteristic of RA and is predictive of radiographic damage to joint tissue in RA.	[66]
Georgiev et al., 2018	Correlation of serum COMP with knee OA: a meta-analysis. The measurement of COMP is a novel knee OA diagnostic. A meta-analysis of nine knee OA studies where serum COMP levels were measured showed consistent significantly elevated serum COMP levels in knee OA patients compared to controls. Meta-analysis showed serum COMP levels could distinguish OA from non-OA patients and were discriminative enough to distinguish between different clinical grades of OA.	[67]
Laudon et al., 2019	Serum COMP levels in individuals who sustained a youth sport-related intra-articular knee injury 3–10 years previously have been shown to display symptoms of knee RA-induced COMP levels and/or COMP degradation. COMP is thus a historical marker of cartilage injury and the pre-history of knee-joint loading and trauma, which both contribute to knee joint OA development.	[57]
Udomsinprasert et al., 2024	Correlation of COMP protein and mRNA levels with histological evidence of damage to knee joint tissues in OA. A recent study has confirmed COMP levels were significantly elevated in serum and synovial fluid of knee OA patients, especially in advanced OA stages, and correlated with radiological severity, body composition, physical performance, knee pain, and disability. COMP mRNA expression is markedly upregulated in the inflamed synovium in knee OA, consistent with immunohistochemical localization of COMP in the inflamed lining and sub-lining layers of knee OA synovium, and positively correlated with COMP levels in OA serum and synovial fluid samples.	[58]

COMP is primarily a protein of the cartilage ECM; however, high levels of COMP also occur in fibrotic scars, systemic sclerosis of the skin and in tendon, with COMP levels elevated in response to physical activity and tissue loading post-injury [68]. COMP also plays a role in vascular wall remodeling and has been found in atherosclerotic plaques [69] and in carotid stenosis [70]. Elevation in COMP expression and TGFβ activity has been observed in Duchenne muscular dystrophy and in fibrotic skin disorders such as keloids and scleroderma. COMP is overexpressed by scleroderma dermal fibroblasts [27]. Widespread mutations in COMP (COMPopathies) cause ER stress and result in chondrocyte apoptosis and diseases where the skeleton is distorted in pseudoachondroplasia (PSACH) [71,72,73] and multiple epiphyseal dysplasia (MED) [24,74]. COMP has also been immunolocalized to the fibrous component of the infrapatellar fat pad [75] in knee joints and is an aggrecan-binding protein mediated through interactions with the glycosaminoglycan (GAG) side chains of aggrecan [76].

### 1.2. COMP Is a Biomarker of Tissue Degradation

COMP is widely associated with multiple diseases and can act as a biomarker for osteoarthritis (OA), rheumatoid arthritis (RA), intervertebral disc degeneration (IVDD), and psoriatic arthritis. Serum levels of circulating COMP have been used as a diagnostic, prognostic indicator of OA disease severity and responses to treatment (reviewed in [1], Table 1). Blood and urine have been used for the detection of COMP levels in the early stages of knee OA [78] and in long-established OA (10–13 year duration) [79]. COMP is a well-established biomarker of knee OA [58], including radiographic trauma-induced knee OA [3]. Furthermore, COMP is cleaved by the proteinase ADAMTS4 (a disintegrin and metalloproteinase with thrombospondin motifs-4) in OA, generating a neoepitope that has found diagnostic application not only in OA [80] but also in other forms of degenerative joint disease arising from articular cruciate ligament damage [81]. ADAMTS-7 and ADAMTS-12 degrade COMP; expression of these enzymes is significantly upregulated in OA articular cartilage and RA synovium [82,83]. Antibodies against ADAMTS-7 or ADAMTS-12 dramatically inhibit TNF-α and IL-1β-induced COMP degradation. Suppression of ADAMTS-7 or ADAMTS-12 expression using siRNA also significantly reduces COMP degradation and the development of OA. ADAMTS-7 and ADAMTS-12 mediated COMP degradation is inhibited by the endogenous serum inhibitor α-2 macroglobulin [84]. Furthermore, granulin-epithelin chondrogenic growth factor disturbs the interaction between COMP and ADAMTS-7 and ADAMTS-12, preventing the degradation of COMP by these enzymes [85]. The major fragment of COMP released following IL-1α stimulation is a 110 kDa fragment that co-migrates in SDS PAGE with a major COMP fragment present in human arthritic synovial fluid samples and a COMP fragment generated by MMP-9 and other MMPs [86,87]. However, *Batimastat* (BB94), a broad-spectrum MMP and ADAM inhibitor, only partially inhibits the generation of this 110 kDa COMP fragment. Thus, a proteinase other than an MMP is responsible for the degradation of cartilage COMP in OA/RA. COMP is cleaved by ADAMTS-4, but not ADAMTS-1 or -5, generating the COMP 110 kDa fragment [80,87]. ADAMTS-7 and 12 also generate this COMP neoepitope [82,83].

Serum COMP measurements have been used to monitor longitudinal cartilage degradation associated with cruciate ligament injury and the development of degenerative joint changes [78]. COMP has well-established roles in ECM stabilization, enhances cellular proliferation and ECM mechanical competence in the growth plate and articular cartilage, ligament, meniscus, and tendon [19], supports the joint lubricative properties of lubricin [88], and thus can assist in joint articulation [24]. COMP fragments in joint tissues are indicative of degradative changes in joint tissues. COMP has been used as a diagnostic and prognostic indicator and as a marker of the arthritis severity and the effect of treatment [1].

### 1.3. Serum COMP Levels in Variably Loaded Skeletal Tissues

Serum COMP levels show some association with OA severity and the loading experienced by joint tissues [89,90,91]. Even moderate walking activity can significantly influence serum COMP levels [91]. In vivo exercise differentially regulates serum COMP concentrations and knee cartilage deformation, leading to changes in COMP distribution due to changes in cartilage volume affected by mechanical and biochemical factors [92,93]. A significant increase in serum COMP levels in the normal knee (mean 41.1 ng/mL) has been observed with changes in the angulation of the knee articulating surfaces in the anterior cruciate ligament reconstructed knee. This apparently reflects cartilage remodeling during the knee reconstruction process but would also be influenced by altered biomechanical microenvironments [94]. However, serum COMP arises from turnover of vascular, ligament and synovial tissues in addition to articular cartilage. Genetic loci affecting serum COMP levels have been identified and suggest serum levels of COMP are likely to occur independently of OA subtypes [95]. Measurement of serum COMP levels is the most useful parameter to monitor in the tracking of early knee OA in ACL deficient knees [96].

#### Animal OA Models

Animal OA models have proved useful in the investigation of the early stages of OA and are considered to offer the most effective therapeutic window where therapeutic interventions that reverse disease pathology are most likely to succeed. Animal models are also useful for longitudinal studies on OA development and identification of biomarkers that are potentially predictive of the development of the OA condition [97]. Associated metabolic changes that occur in joint tissues coordinated with changes in soluble biomarkers of OA disease progression can also be identified in these models and compared with biomarkers occurring in biological fluids with the development of OA in man [97,98,99]. Rabbits [97,98,99,100], mice [101,102], rats [103,104,105], guinea pigs [106,107], dogs [108], sheep [109,110,111] and even horses [112] have all been developed as OA animal models. A number of recommendations have been published on how the histology of joint tissues in these OA models should best be conducted as part of an OARSI histopathology initiative [113,114,115,116,117,118]. Combined detection of serum CS846 and COMP levels has been used for the diagnosis of OA and to monitor OA progression and its severity in a rat OA model [104]. Dual detection of serum CTX-II (C-terminal cross-linked telopeptides of type II collagen) and COMP concentrations is also an effective method for the early diagnosis of OA and evaluation of OA severity in a rabbit OA model [97].

## 2. COMP and Tissue Fibrosis

Skin fibrosis by activated fibroblasts results in altered ECM organization. Excessive matrix deposition due to altered cytokine profiles occurs in pathological conditions such as scleroderma and keloids with excessive dermal collagen deposition. COMP interacts with type I collagen and organizes fibrillar networks in normal healthy skin [119]. COMP deposition is enhanced in the dermis in various fibrotic conditions. COMP levels are significantly increased in fibrotic dermal lesions in scleroderma; this alters the supramolecular architecture of collagen networks in fibrotic skin pathologies [29]. However, in normally healing skin wounds driven by myofibroblasts, COMP is barely detectable. Enhanced COMP expression in skin fibrosis appears due to a particular fibroblast population that is induced by TGFβ and biomechanical forces.

### COMP Interactions with Collagen Networks in Tissue Fibrosis and Cancer

COMP/TSP-5 interaction with type I and type II collagen networks regulates ECM assembly and stabilization [120,121]. Moreover, COMP’s pentameric structure facilitates simultaneous concerted interactions with multiple TGF-β1 molecules, resulting in the sustained activation of the TGF-β signaling pathway [35]. COMP is significantly upregulated and associated with poor survival in colon cancer [121]. This suggests that COMP may be an appropriate therapeutic target to focus on in the treatment of this cancer type.

Pentameric COMP enhances TGF-β-dependent cell signaling by clustering multiple TGF-β1 molecules and receptors at the cell surface, promoting the activation of cell receptors and TGF-β activity. Direct visualization of multivalent binding of TGF-β and BMP-2 with COMP has been achieved using high-resolution atomic force microscopy [34,35]. This reveals the dynamic conformational changes that occur during growth factor binding to COMP. COMP regulates the TGF-β signaling pathway in skin, pulmonary fibrosis and in atrial fibrosis and is a major pathogenetic factor acting in combination with Ang 2 in the development of atrial fibrillation. Protein levels of TGF-β1, P-Smad2, and P-Smad3 are decreased after COMP gene silencing [122], showing that COMP knockdown can inhibit TGF-β activation in atrial cells and this effect is reversed upon re-activation of TGF-β [123]. COMP induces fibrillar collagen-I deposition via CD36 receptor signaling and activation of the MEK1/2-pERK1/2 pathway, and participates in ECM remodeling, contributing to the pathophysiology of liver fibrosis [124].

COMP/TSP-5 interaction with type I and type II collagen networks regulates ECM assembly and stabilization [120,121]. Moreover, COMP’s pentameric structure facilitates simultaneous concerted interactions with multiple TGF-β1 molecules, resulting in the sustained activation of the TGF-β signaling pathway [35]. COMP is significantly upregulated in colorectal cancers and is associated with poor survival [121]. This suggests that COMP may be an appropriate therapeutic target to focus on in the treatment of this cancer type. Colorectal cancer is one of the leading global cancer types. Bioinformatics analysis using an integrated machine learning algorithm has identified roles for COMP in immune-mediated and TGF-β-driven colon fibrosis in cancer progression and established COMP as a hub gene that was significantly upregulated in colon cancer [125], identified cancer sub-types and established roles for infiltrating M2 macrophages in the disease process [126]. Furthermore, this work also showed the potential of TGFβ-targeted anticancer drugs in the treatment of this condition.

Skin fibrosis by activated fibroblasts results in altered ECM organization. Excessive matrix deposition due to altered cytokine profiles occurs in pathological conditions such as scleroderma and keloids with excessive dermal collagen deposition. COMP interacts with type I collagen and organizes fibrillar networks in normal healthy skin [119]. COMP deposition is enhanced in the dermis in various fibrotic conditions. COMP levels are significantly increased in fibrotic dermal lesions in scleroderma; this alters the supramolecular architecture of collagen networks in fibrotic skin pathologies [29]. However, in normally healing skin wounds driven by myofibroblasts, COMP is barely detectable. Enhanced COMP expression in skin fibrosis appears to be due to a particular fibroblast population that is induced by TGFβ and biomechanical forces.

## 3. COMP and Regulation of the Complement System

COMP is the first extracellular matrix protein for which an active role in inflammation has been demonstrated in vivo [127]. It can activate one complement pathway while potentially inhibiting another at the same time. The net outcome of these interactions is most likely determined by the types of released COMP fragments in tissues, which may have feed-back effects on cellular regulation. This may be disease-specific and may antagonize growth factor activities in vivo in different ways.

Small COMP fragments have been identified in synovial fluid, and these serve as biomarkers of cartilage degradation [128]. Levels of these fragments detectable in serum are enhanced in human OA and in animal models of OA pathology [129]. ADAMTS 7 and ADAMTS 10 can degrade COMP; however, this fragmentation process can be prevented by granulin-epithelin precursor [85]. Degradation of COMP by ADAMTS7 and ADAM12 is also inhibited by α2-macroglobulin [84]. MMPs and ADAMTS 4 also degrade COMP, generating a major 110 kDa fragment [86,87].

Proteomic analysis of tendon shows disease stage-specific fragmentation of COMP and differential cleavages with disease severity [130]. Alteration in the mechanical loading of the knee joint influences COMP fragmentation [131], OA and RA cartilages also have elevated COMP fragmentation levels [132]. These COMP fragments were extracted directly from cartilage samples rather than being measured in serum or synovial fluid samples.

### Identification of Specific COMP Fragments in Diseased Tissues

COMP fragments also occur in vascular tissues with the development of atherosclerotic lesions. A specific COMP neoepitope fragment has been identified in such lesions; this may represent a novel biomarker for the detection of symptomatic carotid stenosis [70]. Disease-specific COMP fragments have also been isolated by affinity chromatography of synovial fluids from human patients with RA, OA, or acute knee joint trauma [133]. Separation of these COMP fragments by SDS PAGE and in-gel digestion with trypsin, chymotrypsin, or Asp-N and identification of the generated peptide fragments by mass spectrometry identified 12 different COMP neoepitopes. Furthermore, cartilage explants stimulated with TNF-α and IL-6 could also generate a Ser77 COMP neoepitope identified by mass spectrometry. The cell regulatory properties of these COMP fragments and how they potentially effect cartilage degradation have yet to be ascertained; however, by analogy with other cartilage matricryptic peptides, these are liable to have distinct biological activities, as outlined in Table 2.

A COMP neoepitope has also been identified in horses; this neoepitope, detected by ELISA, was elevated in synovial fluid samples from horses with acute lameness and may be useful as a biomarker of early molecular changes in articular cartilage associated with OA [247]. COMP fragments were purified from synovial fluids of horses with intra-thecal tendon injuries and from equine tendon explant media samples and identified by mass spectrometry. A 100 kDa COMP fragment was prominent in diseased tendon and was used as immunogen to raise specific antibodies in rabbits. ELISA demonstrated a 10-fold rise in the mean neoepitope levels for tendinopathy cases compared to controls (5.3 ± 1.3 µg/mL (*n* = 7) versus 58.8 ± 64.3 µg/mL (*n* = 13); *p* = 0.002) [248].

## 4. COMP and TGF-β Signaling

Pentameric COMP enhances TGF-β-dependent cell signaling by clustering multiple TGF-β1 molecules and receptors at the cell surface, promoting the activation of cell receptors and TGF-β activity. COMP regulates the TGF-β signaling pathway in skin, pulmonary fibrosis, and, in atrial fibrosis, is a major pathogenetic factor acting in combination with Ang 2 in the development of atrial fibrillation. Protein levels of TGF-β1, P-Smad2, and P-Smad3 are decreased after silencing COMP, showing that COMP knockdown can inhibit TGF-β activation in atrial cells, and this effect is reversed upon re-activation of TGF-β [123]. Smad stands for Caenorhabditis elegans SMA (“small” worm phenotype) and MAD family (“Mothers Against Decapentaplegic”). COMP induces fibrillar collagen-I deposition via CD36 receptor signaling and activation of the MEK1/2-pERK1/2 pathway, and participates in ECM remodeling, contributing to the pathophysiology of liver fibrosis [124].

### Identification of Biomarker Proteins Including COMP in Arthritis Sub-Types

In a recent comprehensive knee OA proteomics study [249], out of 333 quantified proteins identified, including COMP, 45 proteins were differentially expressed in OA versus control tissues and significantly altered in OA compared to RA, confirming COMP’s strong diagnostic and discriminative potential [250]. Analysis of COMP levels differentiated OA from RA, with a synovial fluid COMP concentration of up to <3136 ng/mL noted in some OA samples. COMP levels were significantly higher in OA males compared to female OA samples and may be sex-linked to an estrogen decline with aging and cartilage degradation.

## 5. The Bioregulatory Roles of Matricryptins/Matrikines in Tissue Homeostasis: Emergence of COMP Fragments as New Matricryptin Members

Matricryptins [251] are bioactive peptide fragments released from ECM proteins, such as collagens and proteoglycans. These fragments have been shown to regulate angiogenesis, cancer, fibrosis, inflammation, neurodegenerative diseases and wound healing [252]. Endostatin, the C-terminal noncollagenous NC1 domain of type XVIII collagen, is generated as 24–30 kDa peptide modules by cleavage in the protease-sensitive hinge region between the trimerization and endostatin domains of collagen XVIII NC1 by MMP-3, -7, -9, -13 and -20 [191,253]. Collagen XIX, an atypical, non-fibrillar collagen of basement membrane, has roles in the formation of parvalbumin positive inhibitory synapses [254]. A proteolytically released collagen XIX matricryptin fragment interacts with integrin receptors to promote the assembly of inhibitory nerve terminals. Inhibitory synapses comprising ∼20% of the total synapses play essential roles in controlling neuronal activity in the mammalian brain. Disruption in inhibitory synapses is associated with schizophrenia and epilepsy. Loss of collagen XIX results in a reduction of telencephalic PNNs and reduced levels of aggrecan in the PNNs [255]. Coll XIX KO mice display a widespread upregulation of extracellular proteases, which may result in a loss of collagen XIX and aggrecan in schizophrenia and epilepsy PNNs. Collagen XVIII endostatin generated by cerebellar Purkinje cells signal through α3β1 integrins. Association of endostatin with α3β1 integrins aids in the organization of brain synapses [253]. Specialized CNS/PNS basement membranes in the blood–brain barrier, cerebrovasculature, NMJ, perineuronal nets, perisynaptic axonal coats and neuronal synapses, aid in the compartmentalization of the brain ECM, providing unique environments conducive to optimal activity of neuronal cell populations [256]. Basement membrane components are susceptible to degradation by MMPs, leading to the release of matricryptic fragments, some of which have interesting biological properties of potential utility in repair biology. Matricryptins regulate wound healing, fibrosis, inflammation, angiogenesis, and cancer and are involved in infectious and neurodegenerative diseases [251,252,256,257].

### COMP and Complement in Arthritic and Inflammatory Disorders

A number of SLRP proteoglycan family members modulate the Complement system. Decorin and biglycan both strongly inhibit C1q binding to human endothelial cells and pro-monocytic U937 cells, suppressing C1q-induced MCP-1 and IL-8 production by human endothelial cells and downregulating proinflammatory effects mediated by C1q [258]. The Complement system is highly expressed in OA, RA and inflammatory arthritis [259,260]. Proteomic and transcriptomic analyses of synovial fluids and membranes from individuals with OA have demonstrated that expression and activation of complement is abnormally high in human OA joints, suggesting it has a central role to play in the pathogenesis of OA [127,261].

Fibromodulin, Osteoadherin, and biglycan display Complement-modulatory activity that enhances the killing of *Moraxella catarrhalis* in respiratory disorders, defining a new antibacterial protective role for SLRPs in the bioregulation of the Complement system [262]. Fibromodulin binds directly to C1q and activates the classical Complement pathway. Osteoadherin, like fibromodulin, also binds C1q and activates the classical pathway strongly while moderately activating the terminal pathway. SLRPs regulate Complement activation in ECM diseases characterized by chronic inflammation such as RA, atherosclerosis, OA and COPD [263]. In contrast, decorin and biglycan act as inhibitors of activation of the Complement cascade, cellular interactions, and proinflammatory cytokine production mediated by C1q. These two proteoglycans are likely to downregulate proinflammatory effects mediated by C1q, and possibly also the collectin collagen-containing C-type lectins that constitute part of the innate immune system operative in inflammatory diseases [258].

Several ECM components also contain anti-angiogenic peptide modules with a diverse range of bioregulatory properties, as shown in Table 2. A range of bioactive peptides have been identified in fibronectin, laminin and plasminogen with diverse bioregulatory properties, ranging from promotion of active MMP release in tissues, cell adhesion, and cell migration to inhibition of angiogenesis, and have found application in a diverse range of tissue engineering strategies in tissue repair. While inhibition of blood supplies to tumors is a useful therapeutic approach to inhibit tumor development, stimulation of new blood vessels in tissue repair is equally important in ECM remodeling and the healing responses in repair biology. The engineered COMP-Ang 1 and 2 chimeric proteins described later in this review are particularly powerful angiogenic stimulatory proteins with the potential to significantly improve repair responses in a range of tissues.

COMP is also degraded in disease processes, generating a number of peptide fragments. These are useful as biomarkers of disease progression and severity in arthritic disorders, tendinopathy and vascular disease (Table 2). Further studies will more fully characterize the biological properties of individual COMP fragments with time. Identification of COMP fragments is a relatively recent achievement; most of the biological properties of matricryptic peptides has been identified over the last two decades. Based on the range of bioregulatory properties so far identified for matricryptic peptides, COMP peptides are expected to also have novel properties in bioregulation, with the likelihood to improve wound healing responses of applications in repair biology. Ongoing studies with COMP peptide modules are expected to uncover novel roles for these molecules in pathobiology. COMP peptide fragments are new members of the matricryptin/matrikine family of bioactive peptides. Preliminary studies show these COMP fragments are differentially produced in different diseases and are useful additions to COMP as biomarkers of tissue degeneration. Examination of the diverse biological properties of the matricryptins presented in Table 2 suggests that interesting properties may also be uncovered for the COMP peptides relatively recently described.

Proteoglycan and proteoglycan fragments have been used as biomarkers for several diseases characterized by dysregulated ECM remodeling in OA, RA, atherosclerosis, thoracic aortic aneurysms, CNS disorders, viral infections, and cancer [264]. After injury, proteolytic degradation of ECM generates bioactive matricryptic fragments, exposing cryptic sites with actions distinct from the parent molecule from which the matricryptin was derived. Matricryptins contribute to the regulation of inflammatory, reparative, and fibrogenic cascades through effects on several different cell types both in acute and chronic settings [265]. Most matricryptins released from collagens and proteoglycans exhibit anti-angiogenic and anti-tumor properties. Controlled proteolysis of ECM components releases bioactive fragments or unmasks cryptic sites in ECM components that play key roles in controlling various physio-pathological processes, including angiogenesis, tissue remodeling, wound healing, inflammation, cell migration and adhesion, tumor growth, and metastasis [251]. Angiogenesis plays a pivotal role in various pathological conditions, making it a key target in therapeutic development. Anti-angiogenic therapies are gaining traction for their potential in treating a range of angiogenesis-dependent diseases. Among these, endogenous angiogenesis inhibitors, particularly endostatin, have garnered significant attention for their therapeutic potential. While extensively studied for its anti-angiogenic effects in cancer, endostatin also exhibits anti-atherosclerotic and anti-fibrotic properties [266]. Perlecan and type XVIII collagen also contain C-terminal anti-angiogenic modules (Table 2).

## 6. COMP Has Roles in Malignancy, Cardiovascular Diseases, and Tissue Fibrosis

**Figure 3:**
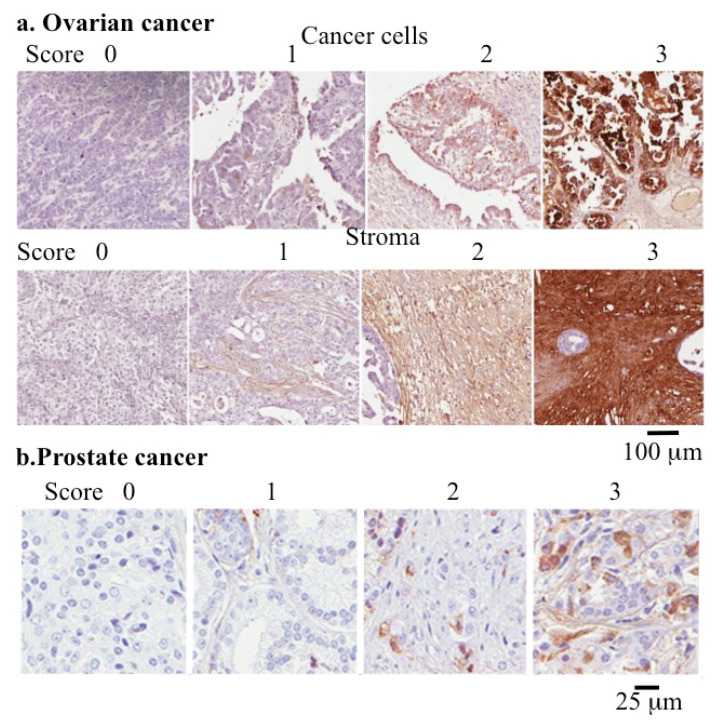
Use of COMP as a quantitative cancer biomarker. Immunolocalization of COMP in cancer cell and stromal tissue samples from ovarian cancer (**a**) and prostate cancer (**b**) used to determine the pathological grade of these conditions. Images reproduced from [272,277] under open access Creative Commons Attribution (CC-BY) licenses. Scale bars 100 μm in (**a**) and 25 μm in (**b**).

COMP also has multiple roles in malignancy, cardiovascular diseases, and tissue fibrosis in a range of tissues [37,53,267,268]. COMP promotes cancer stem cell proliferation through activation of Jagged1 and Notch3 signaling. COMP dysregulation occurs in fibrosis [53,124,269,270], cardiomyopathy [70], breast adenocarcinoma [38], and colon [271], ovarian [272] and prostate cancer [72,273] (Figure 3). Gastric cancer is the world’s third leading cause of cancer deaths, and COMP is significantly elevated in gastric cancer, leading to its proposal as a prognostic marker for this condition [274]. The majority of gastric cancers are adenocarcinomas [275,276].

COMP binds to the cell surface and activates the PI3k (phosphatidylinositol 3-kinase)/AKT (protein kinase B)/mTOR (mammalian target of rapamycin) pathway. This is an intracellular signaling pathway important in the regulation of the cell cycle directly related to cellular quiescence. COMP expression provides radiation resistance in non-small cell lung cancer (NSCLC). The PI3k/AKT pathway promotes the repair of breakages in double stranded DNA [278], and this prevents apoptosis occurring in the tumor cell, providing resistance to radiotherapy of tumor cells. COMP binds to CD36, CD47, and a_v_β_3_ and a_v_β_5_ integrins [120], activating the Src and PI3k/AKT pathways, which promote cancer cell proliferation, invasion and metastasis. Inhibition of COMP may thus be a promising therapeutic target in cancer treatments.

Proteomic studies show COMP promotes oxidative phosphorylation and drug resistance pathways [279]. Overexpression of COMP and therapeutic administration of exogenous COMP provide a protective effect in NSCLC against radiation treatment [279]. It may be possible to target COMP to diminish this protective effect by inhibiting the expression of downstream intermediates in the COMP cell signaling pathway.

Overexpression of sirtuin, a potent tumor suppressor, downregulates the PI3K/Akt/mTOR signaling pathway in NSCLC cells. SIRT6 promotes the radiosensitivity of NSCLC and inhibits the development of tumors [280]. Sirtuin 6 is a *stress responsive histone deacetylase and mono-ADP ribosyltransferase* that regulates chromatin structure, modulating transcription factor access effecting gene expression [281]. *Sirtuin 6 is localized to the* nucleus, where it mediates DNA repair, regulates the expression of metabolic genes, and maintains genomic stability, and through these functions, it acts as a tumor suppressor.

## 7. COMP Expression in Skin and Vascular Tissues

COMP is induced in granulation tissue in skin following injury and is present in dermal scar tissue and found in vascular plaques in atherosclerosis [69]. COMP expression is elevated in fibrotic skin pathologies [29,36,38,53,267,268,270,282] such as keloids [28] and is overexpressed by scleroderma dermal fibroblasts [27]. Myofibroblasts also synthesize elevated COMP levels in systemic sclerosis [283], and interaction of COMP with angiopoietin 1 (Ang1) can upregulate COMP synthesis by retinal endothelial cells [284]. COMP is expressed by vascular smooth muscle cells and is a component of atherosclerotic plaques [47,285] and a biomarker of symptomatic carotid stenosis [70]. Engineered chimeric COMP-Ang1 and COMP-Ang2 proteins show promise as angiogenic agents to improve angiogenesis in tissue repair [51,52]. COMP-Ang1 accelerates muscle regeneration [286]. COMP also interacts with integrin receptors α5β1 and αVβ3 to mediate cell adhesion [287].

## 8. COMP Activates the Complement System

The Complement system is central to the innate immune response and is critical to host defense against pathogens, aiding in the removal of pathogens and dying cells [288,289,290]. The Complement system consists of more than 50 soluble and membrane-bound proteins that have roles to play in tumor progression and metastasis, which are hallmarks of cancer [291,292]. COMP regulates the Complement system and is involved in both the development of and defense against cancer. Complement component 1q (C1q), initiator of the classical complement pathway, binds to COMP [127] and activates the alternative complement pathway [127,293]. The G3 domain of aggrecan also activates the Complement system [294], providing defense against foreign pathogens and acting as a danger sensor, aiding in the removal of dying cells, immune complexes and misfolded proteins [295]. During cartilage turnover, cartilage proteins are fragmented and released into the synovial fluid, where they interact with Complement and are cleared from the synovial fluid to prevent establishment of pro-inflammatory conditions in the joint.

## 9. Mutations in COMP Impact Tissue Organization and Function

**Figure 4:**
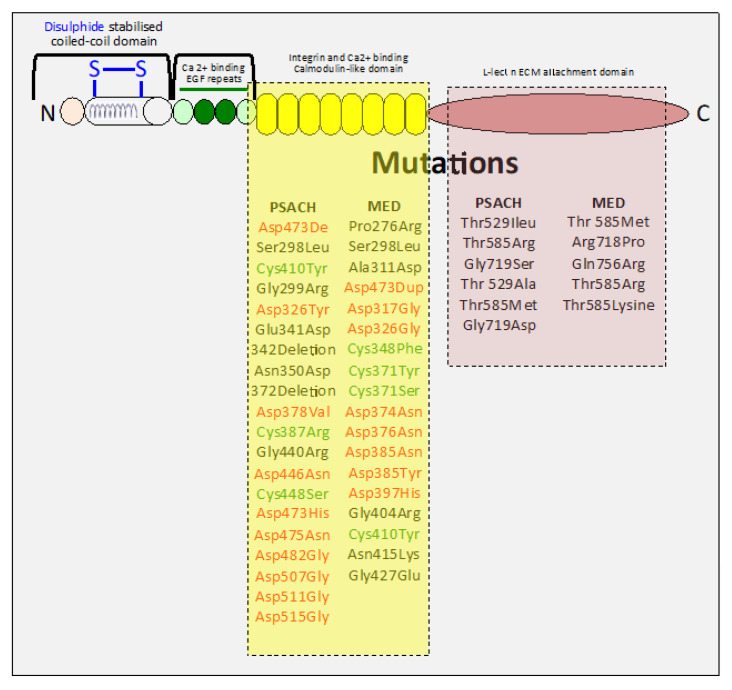
Schematic depiction of COMP showing its structural domains and the mutations in COMP that lead to pseudochondroplasia (PSAH) and multiple epiphyseal dysplasia (MED) during skeletal development. These mutations are predominantly confined to the COMP C-terminal domains, key functional regions of the COMP molecule. PSAH and MED have prominent mutations in the highly conserved aspartate or cysteine residues of the calmodulin-like repeat (CLR) C-terminal domain of COMP, while mutations in the lectin-like ECM attachment C-terminal domain are less numerous.

Mutations in COMP cause pseudoachondroplasia [73], a severe dwarfing condition associated with premature joint degeneration and significant lifelong joint pain and less severe multiple epiphyseal dysplasia [72]. A novel COMP variant with health consequences has been shown to induce multiple epiphyseal dysplasia and osteochondritis dessicans [296]. Over 40 frame insertion/deletion mutations in the COMP gene cause the skeletal dysplasias, pseudoachondroplasia and less severe multiple epiphyseal dysplasia [36,297,298,299] (Figure 4). Pseudochondroplasia is almost exclusively caused by COMP mutations, whereas various forms of multiple epiphyseal dysplasia have been attributed to mutations in the genes encoding COMP, type IX collagen COL9A1, COL9A2, and COL9A3chains and matrilin-3 [299]. Two novel mutations, (i) a gross deletion spanning an exon–intron junction exon deletion and (ii) a frameshift mutation resulting in a truncation of the C-terminal domain, have also been identified [299]. The majority of COMP mutations affect the highly conserved aspartate or cysteine residues in the calmodulin-like repeat (CLR) region of COMP. Mutations in the CLR domain produce individuals of severe short stature. Patients carrying mutations within the five-aspartate repeats in amino acids 469–473 in the seventh CLR domain are of extremely short stature. Deletion mutations produce significantly shorter skeletal forms than those in individuals with substitution mutations. A novel mutation in exon 18 of COMP in the C-terminal globular domain has been identified as one producing a severe pseudoachondroplasia phenotype with marked short stature, spinal deformities and deformed skeletal appendages [300]. These results demonstrate unique bouquet-like COMP presentations in the C-terminal domains as key functional regions amenable as molecular templates for the production of engineered proteins with novel functions. Screening for mutations in the COMP gene, which can lead to PSACH and some forms of MED, have been used to aid in the clinical diagnosis and counseling of patients [301].

## 10. Emerging New Areas of COMP Biology

### 10.1. Roles for COMP in Tumor Biology

While COMP has roles in ECM stabilization through interactions with collagen in normal tissue development, a few studies have recently emerged showing COMP also has roles in tumor development in a number of cancers [302], including prostate [303] and ovarian cancer [272] (Figure 3). These provide tumor cells with a resistance to treatment using chemotherapy and radiotherapy [278,279]. COMP also has immunomodulatory properties in gastric and esophageal adenocarcinoma [272]. COMP has roles in the development of hepatocellular [304,305,306], colorectal [126,271,307], adenocarcinoma [38], urothelial [308], adenoma [309], gastric [274] and breast [37] cancer. Elevated COMP expression levels correlate with poor prognosis. COMP has been proposed as a diagnostic biomarker for these conditions.

### 10.2. COMP in Vascular and ECM Remodeling

COMP expression by vascular smooth muscle cells and interaction with Ang-1 results in sustained vascular remodeling [310,311,312,313]. COMP is also associated with ECM remodeling in tensional and weight bearing tissues, and its expression is modulated by mechanotransduction [19,20,21,22,25,314]. The bridging properties of COMP aid in the spatial organization of collagen fibrils, allowing COMP to contribute to ECM remodeling events in tendon, ligament and cartilage [314]. When these tissues are overloaded, they shed characteristic biomarker fragments of COMP.

### 10.3. COMP-Mediated TGF-β Signaling and Tissue Fibrosis

COMP expression by myofibroblasts can be modulated by TGF-β [269]. COMP is a constitutive component of healthy human skin but is strongly induced in fibrosis binding to collagen I and XII. COMP also promotes the efficient secretion of collagens for assembly of ECM structures [315]. Regulation of TGF-β signaling by COMP in the aorta can lead to atrial fibrosis. COMP knockdown inhibits the activation of the TGF-β pathway, leading to atrial fibrosis and atrial fibrillation [123]. COMP is also a biomarker of tissue fibrosis in Duchenne muscular dystrophy [267] and participates in fibrotic changes in hepatocellular carcinoma [124,268,270,316,317]. Mesenteric fibrosis occurs in small intestinal neuroendocrine tumors. COMP has established roles as a biomarker of the pathologic status of articular cartilage in disease processes such as OA and RA [54,64,67]. Multiple tissues can contribute to serum COMP levels; thus, COMP lacks specificity as a cartilage-specific biomarker. COMP also has emerging roles in fibrotic tissues [124,269,315], vascular and ECM remodeling [18,19,310,311,312,313,314] in traumatized tissues and in tumor biology [126,302,304,307].

## 11. Roles for the Coiled-Coil COMP Domain in Tissue Organization/Stabilization

The coiled coil (CC) motif is common in cytoskeletal motor proteins, transcription factors and attachment protein receptors [318,319,320]. This structural motif involves 2–7 alpha-helices coiled together in dimeric or trimeric rope-like structures [321,322]. Soluble N-ethylmaleimide attachment protein receptor (SNARE) proteins contain an α-helical coiled-coil SNARE motif [318]. CC motifs occur in 5–10% of proteins and have a variety of functions [323] in protein–protein interactions with important biological processes, such as gene regulation by transcription factors; notable examples include the c-Fos and c-Jun oncoproteins. The highly interactive functional muscle protein tropomyosin also utilizes CC motifs to facilitate multiple interactions in muscle [323]. Spectrin, a large, cytoskeletal, heterodimeric cell shape determining protein, contains CC motifs that maintain the stability and structure of the cell membrane and cell shape [171,324,325]. These CC domains have been described as “cellular velcro” that holds together assemblies of molecules and subcellular structures [320]. Of particular importance are CC-mediated protein–protein interactions in gene regulation through transcription factors and receptor kinases in cell signaling pathways.

The CC domain of COMP is a particularly fascinating and useful molecular template in protein engineering. Pentameric chimeras have been developed containing bioactive molecules using the CC domain [51,52]. These have enhanced stability and improved functional properties in the activation or inhibition of specific cell signaling pathways of relevance to repair biology. Activation of the tyrosine kinase with immunoglobulin and epidermal growth factor homology domain 2 (Tie2) receptor by an engineered angiopoietin-2 pentamer has been used to develop a potent stimulatory molecule with an enhanced ability to stimulate angiogenesis in wound repair [312]. The TSP-5/COMP pentamerizing CC has considerable potential in the development of high-affinity stable ligands suitable for clinical or bioengineering applications in the repair of pathological tissues.

Intravitreal angiopoietin-1 combined with the short CC domain of COMP delivered by adeno-associated viral serotype 2 (AAV2.COMP-Ang1) following the onset of vascular damage can rescue or repair damaged vascular beds and attenuate neuronal atrophy and dysfunction in the retinas of aged diabetic mice [326].

Tie2 mediates vascular stabilization and ameliorates neovascular age-related macular degeneration. Mice that received subretinal injections of AAV2.COMP-Ang1 underwent a significant reduction in VEGF levels (29–33%, *p* < 0.01) and choroidal neovascularization volume (60–70%, *p* < 0.01), without decreased levels of HIF1-α. This produced effects similar to those of anti-VEGF agents for the long-term amelioration of neovascular age-related macular degeneration.

Diabetic retinopathy is the leading cause of blindness in the adult population in the USA. Neuroglial and vascular dysfunction in diabetic retinopathy are vision-threatening events that occur in concert, driven by hyperglycemia along a pathway of inflammation, ischemia, vasodegeneration, and blood retinal barrier breakdown. Currently, no therapies exist for normalizing the vasculature in diabetic retinopathy. A single intravitreal dose of AAV2.COMP-Ang1 ameliorates structural and functional disruption in diabetic retinopathy in Ins2Akita mice. Sustained recovery of the retinal vasculature was observed over a 6-month recovery period [327].

Tie2 is activated by an oligo-multimeric complex containing multiple Ang 1 domains that clusters Tie2, phosphorylating the Tie2 kinase domain initiating downstream signaling [328,329,330]. Tie2 is expressed by vascular endothelial cells and hematopoietic cell lineages [328,330]. Ang1 has roles in vascular assembly, maturation, stabilization, and vessel protection during developmental and pathological angiogenesis [330,331]. The pentameric CC domains of COMP have been used to develop multimeric chimeras with Ang1 and Ang2 with potent angiogenic activity [51,332]. COMP-Ang1 enhances DNA synthesis and cell cycle progression in human periodontal ligament cells via Tie2-mediated phosphorylation of PI3K/Akt and MAPKs [333] to activate Tie2-mediated angiogenesis and vascular stabilization [334]. Increased proliferation, differentiation, and migration of stem progenitor cells occurs through Tie2-mediated activation of p38 MAPK and PI3K/Akt signal transduction pathways [335]. Accelerated new bone formation by Tie2 in rat calvarial defects [336] prevents periodontal damage, enhances mandible bone growth [337] and protects against radiation-induced bone marrow damage in mice [338].

Chondrocyte COMP organizes cartilage ECM components and is of potential application in cartilage repair strategies [339]. COMP is immunolocalized to the infrapatellar fat pad [75] and is abundant in cartilage, where it enhances collagen fibrillogenesis [16] and interacts with a number of growth factors [32,34,35,340], ECM proteins and cellular receptors. Infrapatellar fat pad adipose-derived stem cells co-cultured with articular chondrocytes from OA patients exhibited increased chondrogenic gene expression [341]. COMP was distributed across all layers of cartilage undergoing repair using these stem cells and in the calcified cartilage undergoing endochondral bone formation [342]. COMP thus shows promise in cartilage repair biology, particularly in the cartilage bone interface facilitating integration of neocartilage with the underlying bone. COMP levels are elevated in cartilage repair induced in vitro by TGF-β, suggesting COMP promotes repair.

## 12. Therapeutic Opportunities with COMP

Engineered chimeras based on the pentameric CC domain of COMP have potential roles in repair biology. COMP is also a useful diagnostic in several disease processes and has been used to monitor the effectiveness of drugs in development to treat these diseases. COMP is thus useful in the assessment of the efficacy of therapeutics in many diseases. As already discussed, COMP is an established biomarker of cartilage destruction [343]; measurement of serum COMP metabolites in RA patients treated with DMARDs has been proposed as a means of evaluating the effectiveness of these drugs [344]. Serum COMP levels are significantly elevated in patients with active RA compared with control subjects [345,346,347]. COMP levels have also been used to evaluate the progression of OA and the efficacy of DMOAD treatment [250,348,349]. COMP has notable functions in the regulation of cellular behavior as a multivalent interactive bridging molecule. This provides a signaling platform for TGF-β and BMP-2 in chondrogenesis, osteogenesis, tissue fibrosis, vascular and ECM remodeling, and cancer.

### 12.1. COMP as a Biomarker of Disease

COMP is a cancer biomarker and is useful in staging the progressive/poor prognosis/cancer remission phases of diseases including colon, prostate, pancreas, stomach and rectal adenocarcinomas, lymphoid neoplasms, diffuse large B-cell lymphoma, and kidney and ovarian adenocarcinomas. Melanoma, testicular germ cell tumors, epithelial tumors of the pre-vascular mediastinum (a space in the chest that holds the heart and other major structures) and uterine carcinosarcoma are also troublesome tumors in urgent need of effective treatments. COMP has been proposed as a prognostic biomarker and potential therapeutic target for the treatment of gastric cancer [274].

Idiopathic pulmonary fibrosis (IPF) is a fatal, rapidly progressive interstitial lung disease with unpredictable clinical outcome. COMP is a potential diagnostic marker for the IPF gene, opening the possibility for novel investigations into pathogenic IPF pathways and therapeutic approaches with COMP, integrating machine learning and neural networks in new diagnostic therapeutic approaches [350].

### 12.2. Application of COMP Ang 1 and COMP Ang 2 Chimeric Proteins in Repair Biology

Angiopoietin 1 (Ang 1) is a specific growth factor that generates a stable, mature vasculature through the Tie2 (angiopoietin-1 receptor, tyrosine-protein kinase receptor) TEK)/PI3K/AKT cell signaling pathway, which has important roles in skeletogenesis and the maintenance of mature tissue functional properties. Ang 1 also promotes LYVE-1 positive lymphatic vessel formation [351]. A COMP-Ang 1 chimeric protein has been prepared with properties superior to those of Ang 1 in isolation, for the phosphorylation of the Tie2 receptor and AKT (Protein kinase B) in the stimulation of angiogenesis [51]. COMP-Ang 1 provides a multimeric platform with the pentameric COMP structure facilitating interactions with TGF-β and BMP-2. The COMP multimeric environment is important for growth factor-receptor activation and growth factor interactions that promote cellular proliferation. Receptor clustering is a well-established activation phenomenon in effective cellular transmembrane signal activation [351,352]. COMP-Ang 1 interaction with BMP-2, ascorbic acid and beta glycerophosphate stimulates osteoblast proliferation and the formation of bone by interaction with alkaline phosphatase. BMP2 and COMP-Ang 1 have been proposed as useful components for fracture repair in destructive bone diseases [45]. COMP-Ang 2 chimeric proteins have also been prepared. COMP-Ang 2 strongly promotes endothelial cell survival, migration, and capillary tube formation in a Tie2-dependent manner. The potency of COMP-Ang 2 is almost identical to that of COMP-Ang 1 [51]. Different oligomeric Ang 2 constructs have also been prepared by replacement of the amino-terminal domains of Ang 2 with dimeric, tetrameric, and pentameric short coiled-coil domains derived from GCN4, matrilin-1, and COMP. GCN4 is a transcriptional activator in the bZIP family that regulates amino acid biosynthetic genes in the yeast Saccharomyces cerevisiae and is a master regulatory transcription factor for gene expression in yeast [353].

COMP-Ang 2 strongly binds and activates Tie2, whereas GCN4-Ang 2 and MAT-Ang 2 bind weakly or with moderate affinity and activate Tie2. Although native Ang 2 strongly binds to Tie2, it does not activate Tie2; however, the multimeric environment provided by COMP pentameric Ang 1 or Ang 2 constructs promotes this activation process. COMP-Ang 1 and BMP-2 have been administered to critical sized calvarial defects in mice using absorbable collagen sponges. This resulted in recruitment of pericytes into the defect site, osteoblast proliferation and an elevation in osteoblast-specific gene expression for bone sialoprotein, osteocalcin and osterix and phosphorylation of Smad/1/5/8, resulting in formation of mineralized bone in the defect site [354]. COMP-Ang 1 thus has therapeutic value in bone repair applications [52].

## 13. Concluding Remarks

The CC domain of COMP is a key domain in its pentameric structure, spatially organizing it as a bridging structure in a highly effective multimeric interactive platform for cell receptors and growth factors. The CC structure is an interesting molecular template that has been used to engineer stable chimeric proteins with multimeric functional ligands that aid in the activation of cellular receptors. An engineered COMP-Ang 2 chimeric protein is a highly effective angiogenic molecule that shows significant potential in wound repair [312]. Multimeric interactions of COMP with BMPs and TGF-β promote osteogenesis and ECM remodeling, although in some cases, this may result in tissue fibrosis. This emphasizes the need to fully understand interactive processes with COMP. Notwithstanding this, COMP shows a diverse range of interactive properties in a range of biological processes requiring further exploration; COMP is thus much more than a mere marker of tissue pathology. COMP is useful in diagnostic applications that can support drug developments for disease processes and shows promise as an adjunctive supportive agent in remedial cancer therapy. COMP thus offers exciting possibilities in biotherapeutics. These applications may offer advanced therapeutic opportunities for problematic clinical conditions.

Poor wound healing in traumatized tissues after surgery, acute illness, or chronic disease impacts the welfare of millions of people worldwide each year and is exacerbated by dysregulation or the poor performance of key components in the tissue repair response [355,356]. Tissue repair is impacted by inflammation, angiogenesis, matrix deposition, fibrosis and cellular recruitment. COMP is a multifunctional protein with roles in all of these processes, as shown in this review. COMP offers considerable potential, through engineered chimeric proteins, in the improvement of tissue repair responses. Applications of such COMP proteins in repair biology are anticipated in the near future.

## Figures and Tables

**Table 2:** Examples of bioactive matricryptin/matrikine fragments of ECM components.

Native Molecule	Fragment	Bioactivity of Fragment(s)
Aggrecan	32 mer peptide Metstatin (BH-P) Recombinant 42 mer peptide MMP and ADAMTS generated aggrecan fragments	The 32-mer fragment is a TLR-2 ligand with the potential to accelerate cartilage destruction in vivo and drives OA-associated pain through TLR-2 [134]. Has anti-anabolic, pro-catabolic, and pro-inflammatory properties in vitro [135]. BH-P angiogenesis inhibitor peptide is of interest as a tumor growth inhibitor [136]. Cartilage aggrecan is cleaved by a wide range of MMPs and ADAMTS enzymes [137], with the IGD being a key cleavage site since this disaggregates aggregan aggregates with HA. A range of neo-epitope antibodies identify these cleavage sites, which occur in a number of pathological tissues [138,139].
Link protein	Link N 16 amino acid N-terminal peptide DHLSDNYTLDHDRAIH	MMPs cleave at two sites in link protein. Stromelysins-1 and -2, gelatinases A and B and collagenase cleave between His16 and Ile17, and matrilysin, stromelysin-2 and gelatinase A cleave between Leu25 and Leu26 [140]. This N-terminal 16 amino acid peptide (Link N) displays growth factor activity, promotes collagen and aggrecan synthesis by chondrocytes, IVD cells and stem cells, and decreases inflammation and tissue fibrosis, promoting IVD and cartilage repair/regeneration [141,142,143,144,145,146,147].
Brain link protein (Bral-1) HA binding peptide	PEP-1 12 mer GAHWQFNALTVR HA binding peptide	PEP-1 is an antagonist HA binding peptide that inhibits neutrophil homing to sites of inflammation [148]. PEP-1 is also a cell-penetrating peptide with the ability to translocate across biological membranes to introduce active cargo proteins and drugs into cells [149]. PEP has been used as a delivery vehicle for drugs and therapeutic proteins into a range of cells in treatment of dense tumor masses [150,151,152,153,154,155,156,157,158,159,160,161,162].
Versican	Versikine	ADAMTS proteases generate a bioactive versican matricryptic fragment containing the N-terminal G1 domain, termed versikine, which promotes inflammation in transitional embryonic tissues [163]. Versikine is a novel bioactive damage-associated peptide active in immune sensing of myelomas and colon tumors [164]. The G1-DPEAAE 80 kDa fragment promotes EMT in developing and diseased tissues, cell migration, proliferation/invasion by tumor and endothelial cells, tumorigenesis and inflammation. Versikine acts in a contrasting manner to intact VCAN in tissues. The 80 kDa G1-DPEAAE fragments promote T cell infiltration and dendritic cell differentiation.
Lumican	Lumcorin LRR9 Chemokine LumC13(C-A)	Lumcorin is a peptide module from the 9th leucine-rich domain of lumican that is an MMP-inhibitor, is anti-angiogenic and has anti-tumor activity [165,166,167,168]. Lumikine is a synthetic peptide based on a C terminal lumican peptide with a cysteine residue replaced with alanine YEALRVANEVTLN. Lumikine binds TGF-β and the type I TGF-β receptor and displays growth factor activity [169,170].
Biglycan	BGN262 peptide, Peniel 2000, Bgm1 biglycan peptide [171] YWEVQPATFR	Biglycan BGN262 [172], equine salivary biomarker for OA sub-chondral bone sclerosis [173], MMP [174], ADAMTS [137] generated and naturally occurring biglycan fragments in OA [175] and IVDD [176,177] and animal models [178,179], in silico designed Peniel 2000 binds and regulates TGF-β1 controlling IVD tissue fibrosis, inhibits IVD degeneration and stimulates IVD repair [180]. Bgm1 modulates TGF-β activity in the IVD [181].
Decorin	Decorunt	Adult human skin contains a truncated form of decorin, termed decorunt, which lacks a portion of the C-terminus of decorin and has a new terminus, VRKVTF [182]. A neoepitope antibody raised to this sequence does not detect full-length decorin. Decorunt has one-hundredth the affinity for type I collage compared to full-length decorin.
Fibromodulin	59, 43, 40, 27 kDa fragments	Fibromodulin is fragmented in OA in cartilage and meniscus with fragments of 27, 40, 43 and 59 kDa detected [175]. Fibromodulin is also fragmented in an ovine model of IVDD [177] and an animal OA model [178]. Fibromodulin is degraded by a range of MMPs and ADAMTSs [137,183]. MMP-13 cleaves N-terminal sulfated tyrosine residues in fibromodulin removing its HS-like interactive properties with growth factors and ECM components [184]. ADAMTS-4 and ADAMTS-5 cleave fibromodulin into 54, 45, 32 kDa fragments in vitro [183].
Perlecan	Endorepellin	Endorepellin is a C-terminal 20 kDa anti-angiogenic peptide module found in domain V of perlecan [185]. Endorepellin is cleaved from the perlecan core protein by BMP-1/Tolloid-like metalloproteases [186]. The activity of endorepellin contrasts with full length perlecan, which is a pro-angiogenic multifunctional proteoglycan [187]. Endorepellin exerts a dual receptor antagonism by engaging with VEGFR2 and α2β1 integrin [188], leading to actin disassembly and blocking of endothelial cell migration required for tube formation and development of new capillaries [189]. This reduces the blood supply to tumors, providing anti-tumor properties.
Collagen XVIII	Endostatin, 20 kDa C-terminal peptide module of collagen XVIII	Endostain is an anti-angiogenic peptide module released from the C-terminus of collagen XVIII by cathepsin L [190] and MMPs [191], which cleave in the protease-sensitive hinge region of the C-terminal domain [192]. Endostatin is essential for vision and RPE function [193]. Reduced endostatin levels in Bruch’s membrane, RPE basal lamina, intercapillary septa, and choriocapillaris may permit choroidal vessel invasion in the retina, where neovascularization exacerbates macular degeneration, leading to impaired vision [194]. Elevated deposition of endostatin occurs in traumatic brain injuries [194].
Agrin	110 kDa C-terminal fragment NT1654, a 44 kDa C-terminal peptide of murine agrin	A C-terminal agrin fragment is a neuromuscular junction-related biomarker of muscle dysfunction and increases with aging in sarcopenia and in other muscle wasting conditions, such as diabetes, COPD, chronic heart failure, stroke, kidney disease and in pancreatic and colon cancer [195,196,197]. NT-1654 is an agrin fragment engineered to prevent cleavage by neurotrypsin. It has acetylcholine receptor clustering activity and the key functions of full-length agrin. Injection of NT-1654 in sarcopenia mouse models with impaired NMJ function results in recovery of muscle performance [198].
Fibronectin	Anastellin	Fibronectin fragments (FN fs) upregulate MMP expression, significantly enhance PG degradation and temporarily suppress PG synthesis, events that are also observed in OA [199,200,201]. FN fs found in OA synovial fluid and generated within cartilage and may contribute to cartilage damage in vivo. However, this FN fs system may also be involved in normal cartilage homeostasis; low concentrations of FN fs enhance chondrocyte anabolic activities [202] and may aid in tissue repair with continuous anabolic effects; low concentrations of FN fs may promote tissue homeostasis. FN fs may thus have cartilage autocrine and paracrine regulatory properties. The MyD88-dependent TLR-2 signaling pathway may be responsible for 29-kDa fibronectin fragment-mediated cartilage catabolic responses [203]. The modulation of TLR-2 signaling activated by DAMPs is a potential regulatory mechanism over cartilage degradation in OA. Anti-metastatic inhibitory fragment of fibronectin is derived from the first FN3 domain [204,205,206].
Laminin	α1 chain IKVAV, AG73 β1 chain YIGSR γ1 chain C16	The laminins are a family of large glycoproteins with important roles in tissue development and stabilization of the basement membrane and ECM. Laminin interactive peptide modules equip it with cell adhesive and matrix stabilizing properties and roles during cell adhesion, migration and tissue development. The laminins have thus been the subject of a number of studies aimed at identifying these bioactive modules [207,208,209,210,211] to determine if synthetic versions of these can be applied in therapeutic tissue repair strategies [212,213,214,215,216,217,218,219,220,221,222]. Four peptide modules have been identified in Laminin-111 of interest in repair biology: IKVAV and AG73 in the α1 chain, YIGSR in the β1 chain and C16 in the γ1 chain [223], and these have potential in tissue repair [219,220,221,222,224,225,226,227,228,229] but in some cases can also promote tumor development [213,214,215,217,218,230,231,232,233,234,235,236,237,238].
Plasminogen	Angiostatin	Human prostate carcinoma cells release enzymatic activity (urokinase) that converts plasminogen to angiostatin. Tissue-type plasminogen activator, or streptokinase, can also generate angiostatin from plasminogen [239]. Angiostatin inhibits angiogenesis in vitro and in vivo and suppresses the growth of Lewis lung carcinoma metastases [240], defining a direct mechanism for cancer-cell-mediated tumor inhibitory activity [241,242]. Angiostatin is a 38 kDa protein that inhibits endothelial cell proliferation [243]. Angiostatin is an internal fragment of plasminogen containing the first four kringle domains. Recombinant kringle 1 and kringle 3 exhibit potent inhibitory activity, while recombinant kringle 2 displays lower inhibitory activity [240]. In contrast, kringle 4 is an ineffective inhibitor of FGF-2-stimulated endothelial cell proliferation. The anti-proliferative activity of angiostatin on endothelial cells is thus shared by kringle 1, 2, and 3, but not by kringle 4 [243]. A more potent inhibitory protein is formed when kringle 4 is removed from angiostatin; Kringle 5 is more potent than angiostatin as an inhibitor of FGF-2-stimulated capillary endothelial cell proliferation [244].
COMP	Fibronectin Hep I and II generated COMP fragments Serum COMP fragments in OA, RA and inflammatory arthritis. COMP fragments in surgically induced OA, collagen-induced arthritis, and TNF transgenic OA animal models. COMP is a major component of the cartilage secretome	Stimulation of equine cartilage explant cultures with N- and C-terminal heparin I and II fibronectin fragments results in chondrolysis and release of COMP fragments into synovial fluid and serum within 24 h. The Hep II fibronectin fragment was more potent than the Hep I fibronectin fragment for the generation of COMP fragments [245]. These COMP fragments are biomarkers of cartilage degradation in OA, RA and inflammatory arthritis [128] representing the early stages of cartilage degradation before disruption of collagen networks. In a study of synovial fluid from 52 OA, 85 RA and 60 patients with inflammatory arthritis, 84% of the RA, 21% of the OA and 60% of the inflammatory arthritis samples had significant amounts of low-molecular-weight COMP fragments (50–70 kDa), and 13% of SF samples taken from patients with RA or inflammatory arthritis were able to degrade COMP in vitro. A novel sandwich ELISA has been developed using an antibody that captures COMP fragments; this reproducibly measures COMP fragment levels in serum of arthritic patients and in surgically induced OA, collagen-induced arthritis, and TNF transgenic OA rodent arthritis models [129]. COMP fragment levels correlated with clinical assessment of the severity of arthritis of OA patients and the progression of surgically induced OA in murine models. This is a useful method for quantitation of serum COMP fragments and to monitor the efficacy of arthritic interventions. In a proteomics study on the secretome of cultured articular cartilage explants, 10 major cartilage peptide signatures of tryptic peptides originating from aggrecan core protein, COMP, fibronectin, fibromodulin, thrombospondin-1 (TSP-1), clusterin, cartilage intermediate layer protein-1, chondroadherin and MMP-1 and MMP-3, were detected [246]. COMP peptides are major components of the cartilage secretome under inflammatory conditions.

## Abbreviations

AAV2	Adeno-associated viruses.
ADAMTS	a disintegrin and metalloproteinase with thrombospondin motifs.
AFM	Atomic force microscopy.
Akt	Protein kinase B.
BMP	Bone mineral protein.
COMP	Cartilage oligomeric matrix protein.
CTX II	C-terminal cross-linked telopeptides of type II collagen.
ECM	Extracellular Matrix.
ER	Endoplasmic reticulum.
GAG	Glycosaminoglycan.
hrAFM	High-resolution atomic force microscopy.
LYVE-1	lymphatic vessel endothelial hyaluronan receptor 1.
MAPK	*Mitogen-activated protein kinase*.
MMP-3	Matrix metalloprotease-3.
mTOR	mammalian target of rapamycin.
OA	Osteoarthritis.
PI3K	Phosphoinositide 3-kinase.
PSACH	Pseudoachondroplasia.
P-Smad	Phosphorylated *Caenorhabditis elegans* SMA (small worm phenotype) and MAD family (Mothers Against Decapentaplegic).
RA	Rheumatoid arthritis.
SIRT6	Sirtuin 6 a stress responsive protein deacetylase/mono-ADP ribosyltransferase.
SLRP	Small leucine repeat proteoglycan.
TSP-5	Thrombospondin-5.
TGF-β	Transforming growth factor-beta.
Tie2	*Endothelial receptor tyrosine kinase receptor-2.*
